# Dysfunctional autophagy induced by the pro-apoptotic natural compound climacostol in tumour cells

**DOI:** 10.1038/s41419-018-1254-x

**Published:** 2018-12-19

**Authors:** Silvia Zecchini, Francesca Proietti Serafini, Elisabetta Catalani, Matteo Giovarelli, Marco Coazzoli, Ilaria Di Renzo, Clara De Palma, Cristiana Perrotta, Emilio Clementi, Federico Buonanno, Claudio Ortenzi, Enrico Marcantoni, Anna Rita Taddei, Simona Picchietti, Anna Maria Fausto, Davide Cervia

**Affiliations:** 1 Unit of Clinical Pharmacology, University Hospital “Luigi Sacco”-ASST Fatebenefratelli Sacco, Milano, Italy; 2 Department for Innovation in Biological, Agro-food and Forest systems (DIBAF), Università degli Studi della Tuscia, Viterbo, Italy; 3 Department of Biomedical and Clinical Sciences “Luigi Sacco” (DIBIC), Università degli Studi di Milano, Milano, Italy; 4 Unit of Clinical Pharmacology, University Hospital “Luigi Sacco”-ASST Fatebenefratelli Sacco, Department of Biomedical and Clinical Sciences “Luigi Sacco” (DIBIC), Università degli Studi di Milano, Milano, Italy; 5 Scientific Institute IRCCS “Eugenio Medea”, Bosisio Parini, Italy; 6 Laboratory of Protistology and Biology Education, Department of Education, Cultural Heritage and Tourism, Università degli Studi di Macerata, Macerata, Italy; 7 School of Sciences and Technologies, Section of Chemistry, Università degli Studi di Camerino, Camerino, Italy; 8 Section of Electron Microscopy, Great Equipment Center, Università degli Studi della Tuscia, Viterbo, Italy

## Abstract

Autophagy occurs at a basal level in all eukaryotic cells and may support cell survival or activate death pathways. Due to its pathophysiologic significance, the autophagic machinery is a promising target for the development of multiple approaches for anti-neoplastic agents. We have recently described the cytotoxic and pro-apoptotic mechanisms, targeting the tumour suppressor p53, of climacostol, a natural product of the ciliated protozoan *Climacostomum virens*. We report here on how climacostol regulates autophagy and the involvement of p53-dependent mechanisms. Using both in vitro and in vivo techniques, we show that climacostol potently and selectively impairs autophagy in multiple tumour cells that are committed to die by apoptosis. In particular, in B16-F10 mouse melanomas climacostol exerts a marked and sustained accumulation of autophagosomes as the result of dysfunctional autophagic degradation. We also provide mechanistic insights showing that climacostol affects autophagosome turnover via p53-AMPK axis, although the mTOR pathway unrelated to p53 levels plays a role. In particular, climacostol activated p53 inducing the upregulation of p53 protein levels in the nuclei through effects on p53 stability at translational level, as for instance the phosphorylation at Ser15 site. Noteworthy, AMPKα activation was the major responsible of climacostol-induced autophagy disruption in the absence of a key role regulating cell death, thus indicating that climacostol effects on autophagy and apoptosis are two separate events, which may act independently on life/death decisions of the cell. Since the activation of p53 system is at the molecular crossroad regulating both the anti-autophagic action of climacostol and its role in the apoptosis induction, it might be important to explore the dual targeting of autophagy and apoptosis with agents acting on p53 for the selective killing of tumours. These findings also suggest the efficacy of ciliate bioactive molecules to identify novel lead compounds in drug discovery and development.

## Introduction

Macroautophagy (hereafter autophagy) targets the delivery of intracellular content to the lysosomal compartments, via the formation of double-membraned vesicles termed as autophagosomes^1–4^. Autophagy is controlled by a set of evolutionarily conserved autophagy-related proteins^5^, occurs at a basal level in all eukaryotic cells, including the unicellular organisms^6^, and is modulated by diverse endogenous systems and cellular stresses^7–9^. The role of autophagy in cell fate is controversial since it may support cell survival, also via suppression of cell death (including apoptosis, necrosis or other forms of non-apoptotic cell death), and activate death pathways^10–12^. Due to the pathophysiologic significance of both malfunction and over-activation of autophagy in different diseases, such as cardiomyopathies, muscular dystrophy, metabolic syndromes, infectious-immune diseases, and neurodegenerative disorders, autophagy has been intensively studied in the past decades^7,8,13^. Noteworthy, as it links cancerous and non-transformed components of the tumour microenvironment, autophagy and its network are important for tumour initiation, progression and response to therapy^14^. In particular, efficient autophagic responses in pre-malignant cells suppress transformation (anti-cancer function) while autophagy supports the natural progression of neoplasms (pro-cancer mechanism). This places the autophagic machinery in the limelight as a promising target for development of multiple approaches for anti-neoplastic agents such as promotion of autophagy for the purpose of cancer prevention and suppression of autophagy as therapeutic intervention in different types of established cancers, including melanoma^12,14,15^.

Natural compounds are involved in the modulation of several cellular events, thus showing a great potential to unravel physiological process and to be translated into clinical use, as for instance cancer treatment^16,17^. Among the bioactive molecules isolated from aquatic eukaryotic microorganisms^18^, we have recently described the cytotoxic and pro-apoptotic effects of climacostol [(Z)-5-(non-2-en-1-yl)benzene-1,3-diol], a natural toxin physiologically produced by the freshwater ciliated protozoan *Climacostomum virens*^19,20^, in tumour cells^18,21–24^. Both in vitro and in vivo evidence demonstrated that climacostol inhibits the viability/proliferation of mouse melanoma cells, induces a persistent inhibition of tumour growth and improves the survival of transplanted mice thus triggering the cell death process as a result of DNA damage and apoptosis^18,23^. The signalling events responsible for the climacostol-induced pro-apoptotic effects rely on the up-regulation of p53 tumour suppressor network that, in turn, activates the intrinsic programmed cell death pathway.

The transcription factor p53 is one of the major barriers against cancer^25,26^. However, the exact mechanisms by which p53 mediates tumour suppression are not understood. Whereas p53-dependent modulation of apoptosis appear crucial for p53-mediated tumour suppression in some studies, in other ones their involvement in p53 effects may be dispensable^27^. Experimental evidence reports that p53, depending on its localisation and mode of action, can act as either an activator or an inhibitor of autophagy^25,28^. Although the extent to which autophagy regulation determines cell death/survival by p53 is unclear, it may underlie key aspects on the biology and treatments of cancer^25,28–31^.

A large number of natural products are involved in autophagy modulation through multiple signalling pathways and transcriptional regulators^32^. In this context, we investigated here how climacostol regulates autophagy through both in vitro and in vivo approaches, as well as the involvement of p53-dependent mechanisms and their impact on autophagosome turnover and cell fate.

## Results

### Climacostol disrupts autophagy in mouse melanoma: in vivo and in vitro evidence

The activity of climacostol in vivo was described in a melanoma allograft model, the B16-F10 cells injected subcutaneously in mice^18,23^. The experimental procedure consisted of 100 µl intra-tumour injections of climacostol at 600 μg/ml or vehicle (control) every 3–4 days for ca. 3 weeks. Using the same experimental paradigm, we defined the autophagy levels in melanomas locally treated with climacostol at day 16. First, we analysed mRNA levels of autophagy mediators by real-time PCR in tumours^1–4^. Transcripts encoding *LC3b*, *p62*, *beclin 1*, *bnip 3*, *bnip 3L*, *atg3*, *atg4*, and *atg5* autophagy genes significantly enhanced in climacostol-treated group (Supplementary Fig. 1a), suggesting a perturbation of autophagic machinery.

**Fig. 1:**
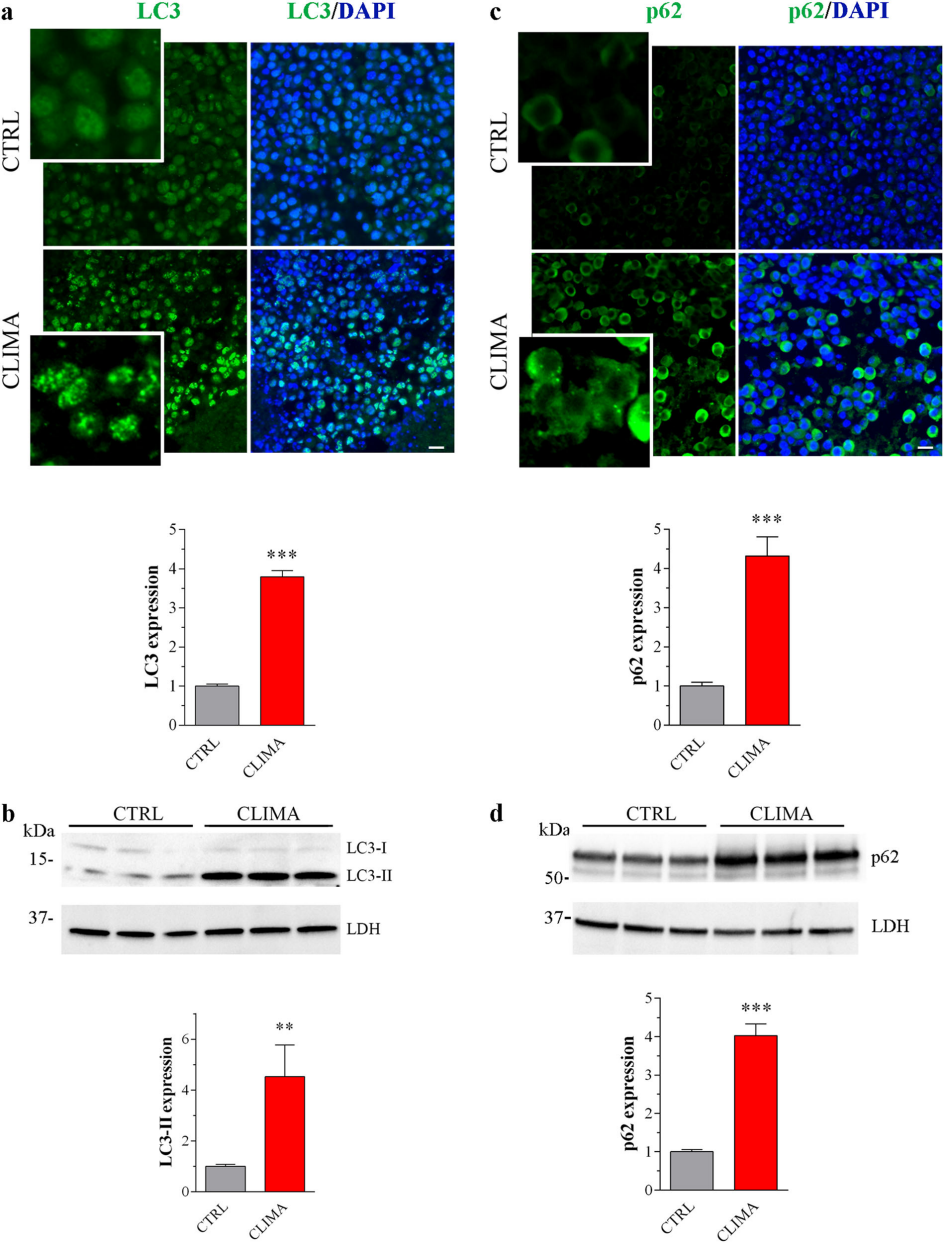
Climacostol impairs autophagy in in vivo melanoma. Subcutaneous B16-F10 melanoma allografts were excised from mice at day 16 of treatment (from day 0 - every 3–4 days) with 100 µl climacostol (CLIMA; 600 μg/ml) or control vehicle (CTRL). **a**, **c** Immunofluorescence imaging of LC3 and p62. DAPI was used for nuclei detection. Scale bar: 50 μm. Inserts represent enlarged image details. Lower panels: quantitative analysis of LC3 and p62 immunofluorescence. A total of 6 different images were analysed per tumour. Results are expressed as fold change of CTRL. **b**, **d** Western blotting images of LC3 and p62 expression. LDH was used as internal standard. Lower panels: densitometric analysis of LC3-II and p62 relative to their respective standard. Results are expressed as fold change of CTRL. Images and data represent the results obtained from 6 animals per experimental group. **p < 0.01 and ***p < 0.0001 relative to CTRL

An increase of LC3 staining and the detection of LC3 puncta, reminiscent of autophagosome formation, was detected in melanoma from climacostol-administered mice, whereas diffuse LC3 staining was visualised in control samples (Fig. 1a). Climacostol treatment also increased lipidated LC3 (LC3-II) levels (Fig. 1b). The lipidation and clustering of LC3 may be the result of both induction and suppression of autolysosomal maturation. The cargo protein p62 is a useful method to distinguish whether autophagosome accumulation is due to autophagy induction rather than an inhibition^3,4^. As shown in Fig. 1c, treatment of B16-F10 allografts with climacostol significantly increased p62 immunofluorescence leading to accumulation of p62-positive aggregates. These results were confirmed by western blot experiments detecting an increase of p62 protein band in climacostol-treated tumours (Fig. 1d).

**Fig. 2:**
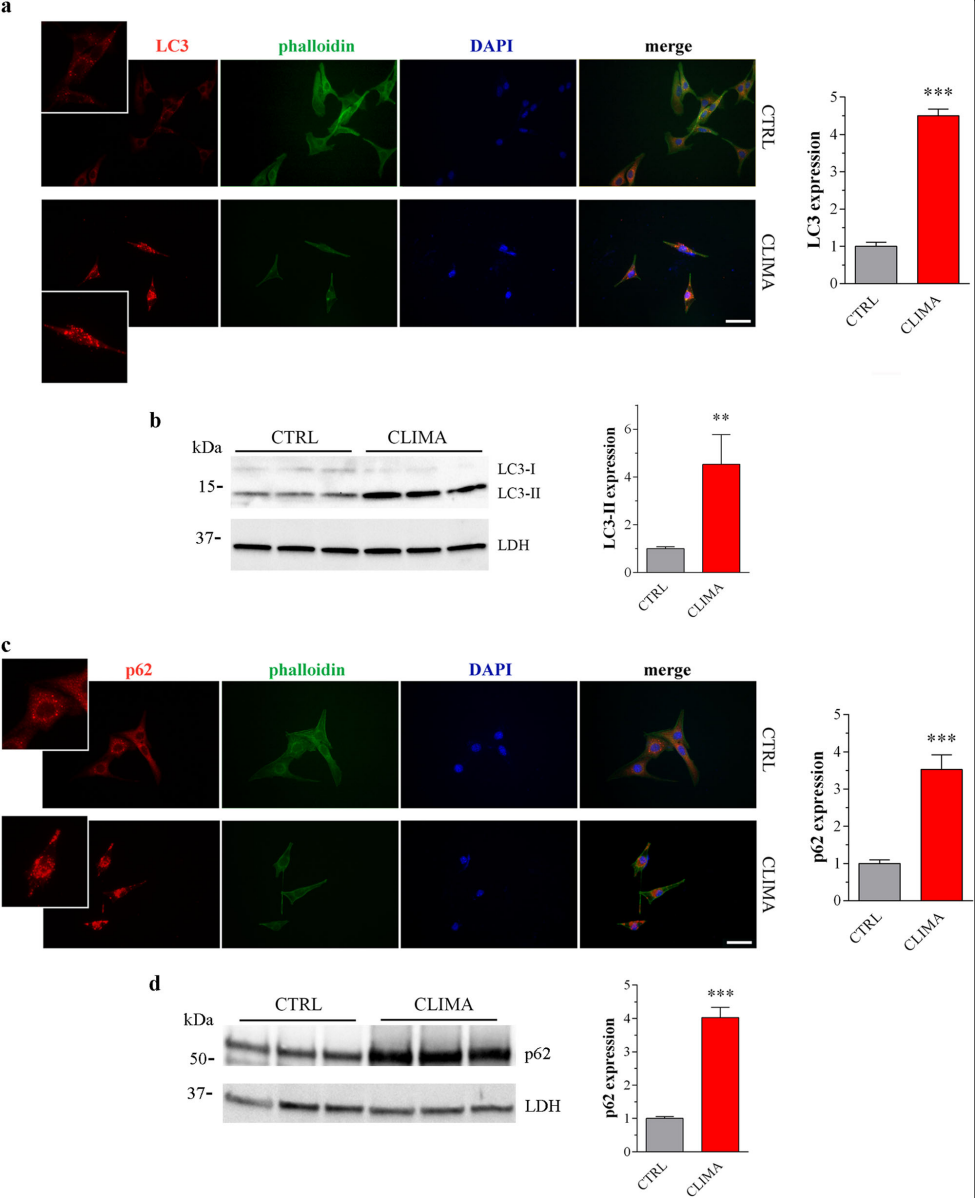
Climacostol impairs autophagy in melanoma cells. B16-F10 cells were cultured in the presence of 30 μg/ml climacostol (CLIMA) or control vehicle (CTRL) for 24 h. **a**, **c** Immunofluorescence imaging of LC3 and p62. Phalloidin and DAPI were used for cytoskeleton and nuclei detection, respectively. Scale bar: 50 μm. Inserts represent enlarged image details. Right panels: quantitative analysis of LC3 and p62 immunofluorescence ( > 30 cells per experimental condition). Results are expressed as fold change of CTRL. Images and data are representative of 6 independent experiments. **b**, **d** Western blotting images of LC3 and p62 expression. LDH was used as internal standard. Right panels: densitometric analysis of LC3-II and p62 relative to their respective standard. Results are expressed as fold change of CTRL. Images and data are representive of 11–15 independent experiments. **p < 0.005 and ***p < 0.0001 relative to CTRL

**Fig. 3:**
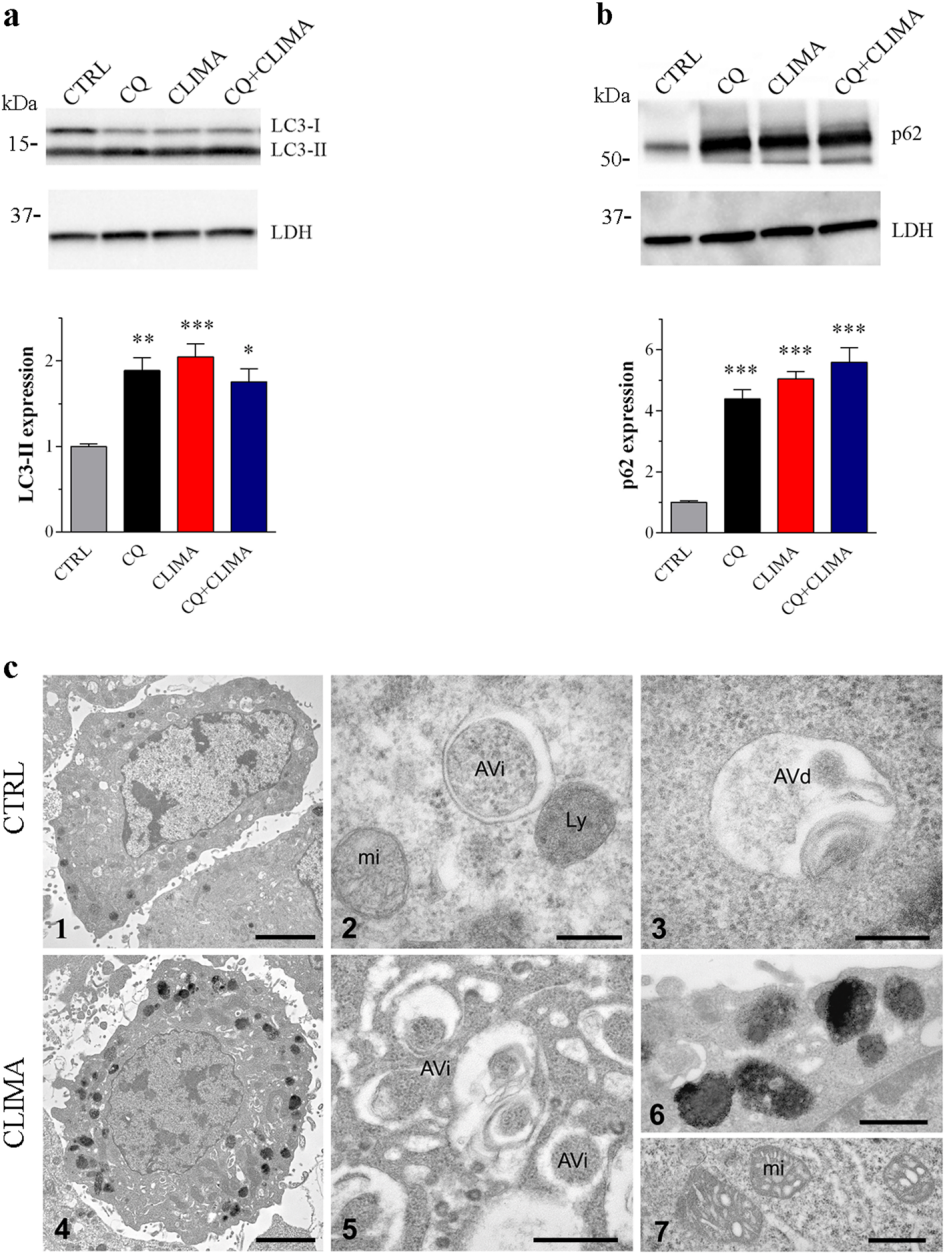
Climacostol impairs autophagic flux. **a**, **b** Western blotting images of LC3 and p62 expression in B16-F10 cells cultured with 30 μg/ml climacostol (CLIMA) or control vehicle (CTRL) for 24 h, both in the absence and presence of chloroquine (CQ; 10 μM, 6 h). LDH was used as internal standard. Lower panels: densitometric analysis of LC3-II and p62 relative to their respective standard. Results are expressed as fold change of CTRL. Images and data are representative of 3–5 independent experiments. *p < 0.05, **p < 0.005 and ***p < 0.0001 relative to CTRL. **c** Electron microscopy images presenting ultrastructure of B16-F10 cells cultured with 30 μg/ml CLIMA or CTRL for 6 h. The panels 1–3 depict representative control cells at increasing magnifications: (1) whole cells; (2) an early or initial autophagic vacuole (Avi), containing morphologically intact ribosomes. The electron-lucent cleft between the two limiting membranes is visible. A dense lysosome (Ly) is also found in contact with the outer limiting membrane of the autophagosome and a normal mitochondria (mi); (3) a late or degradative autophagic vacuole (Avd) containing partially degraded cytoplasmic material. The panels 4–7 depict representative climacostol-treated cells at increasing magnifications: (4) whole cells showing abundant black melanosomes; (5) note the presence of numerous autophagosome-like compartments in the cytoplasm; (6) higher magnification of melanosomes and (7) mitochondria with swollen cristae. Scale bars: 1 and 4: 2 μm; 2 and 3: 200 nm; 5, 6 and 7: 500 nm. Images are representative of 3 independent experiments

B16-F10 cells were treated in vitro with climacostol at its target dose for potency and efficacy (30 μg/ml), inducing cytotoxic, anti-proliferative and pro-apoptotic effects^18,23^. Similarly to in vivo results, 24 h climacostol treatment increased immunofluorescence intensity and puncta of LC3 (Fig. 2a) and LC3 cleavage (Fig. 2b). In addition, we observed higher levels of aggregated p62 and a significant increase of p62 staining in climacostol-treated cells (Fig. 2c) which paralleled with an accumulation of p62 immunoblot levels (Fig. 2d). The effect of climacostol on autophagy was then evaluated by treating cells with the known autophagic flux inhibitor chloroquine (CQ)^8^. B16-F10 cells treated with CQ (10 μM, 6 h)^33^ showed an increased amount of lipidated LC3 and accumulation of p62 (Fig. 3a, b). Of notice, 24 h climacostol-induced accumulation of LC3-II and p62 was not modified in the presence of CQ. The absence of an additive effect between CQ and climacostol is consistent with the inhibition of the autophagic flux exerted by climacostol.

The autophagic response was then analysed using transmission electron microscopy. As shown in Fig. 3c, B16-F10 cells treated with climacostol for 6 h showed accumulation of autophagosomes in the cytosol. Autophagosomes, also referred to as initial autophagic vacuoles (AVi), have been defined as a double-membraned structure containing undigested cytoplasmic contents^4^. The parallel membrane layers (bilayers) of AVi are separated by a relatively narrower or wider electron-translucent cleft, sequestering cytosol, mitochondria, or endoplasmic reticulum membranes not yet degraded. Differently, late or degradative autophagic vacuoles (AVd)^4^, defined as a hybrid organelle generated by the fusion of an autophagosome and a lysosome were clearly detectable in control but scarce in climacostol-administered cells (Fig. 3c). The AVd showed a single membrane and contained materials at various stages of degradation, visualised as intense, dark structures within the vacuoles^4^. Climacostol-treated cells moreover showed disorganised structures, swollen cristae in mitochondria and accumulation of melanosomes in the cytoplasm.

**Fig. 4:**
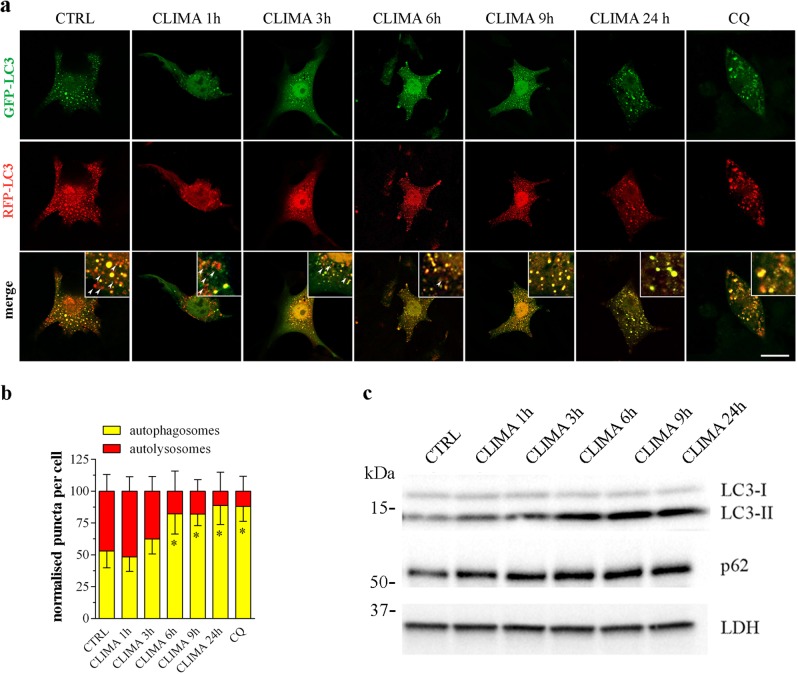
Climacostol impairs autophagic flux. B16-F10 cells were cultured with 30 μg/ml climacostol (CLIMA) or control vehicle (CTRL) for increasing times. Chloroquine (CQ; 10 μM, 6 h) was also used. **a** Confocal fluorescence imaging of cells transiently transfected with mRFP-GFP-LC3 plasmid. This tandem fluorescent-tagged LC3 reporter results in both green (GFP) and red (RFP) fluorescence: autophagosomes appear yellow (with green and red fluorescence) and autolysosomes as only red vacuoles. Scale bar: 30 μm. Inserts represent enlarged image details highlighting the presence of red (arrows) and yellow puncta. **b** The number of autophagosomes and the number of autolysosomes in the merged images were counted and the total number of puncta per cell was calculated as percentage (>30 cells per experimental condition). *p < 0.05 relative to CTRL. **c** Western blotting images of LC3 and p62 expression. LDH was used as internal standard. Images and data are representative of 3 independent experiments

B16-F10 cells were transiently transfected with a red fluorescent protein (mRFP)-green fluorescent protein (GFP)-LC3 as a dual-fluorescence pH sensor of autophagic vacuoles in live cells^34^. The expression of this reporter results in both green and red fluorescence and detects autophagosomes (pH neutral) and autophagosome-lysosome fusion (pH acid)^4,34^, as autophagosomes appear yellow and autolysosomes as only red vacuoles, since the low lysosomal pH quenches GFP more quickly than mRFP. In control conditions, about half of autophagic vacuoles had only red fluorescence signal while the other half had yellow signal (Fig. 4a, b). After treatment of the cells with climacostol, yellow punctate fluorescence significantly increased whilst only-red puncta markedly decreased, indicating a time-dependent blockade of autophagosome maturation/autophagosome-lysosome fusion. The effect of climacostol was detected at 3 h (although below the statistical significance) and reached the almost maximal effect already at 6 h. Close to 90% of the autophagic vacuoles had yellow signals following 24 h treatment. Similar results were observed with CQ alone (10 μM, 6 h). The kinetics of climacostol was further confirmed by immunoblot experiments showing evident LC3 cleavage and p62 accumulation induced by 6 h of treatment (Fig. 4c).

### Climacostol induces cell death/apoptosis and impairs autophagy in human and murine tumour cells

**Fig. 5:**
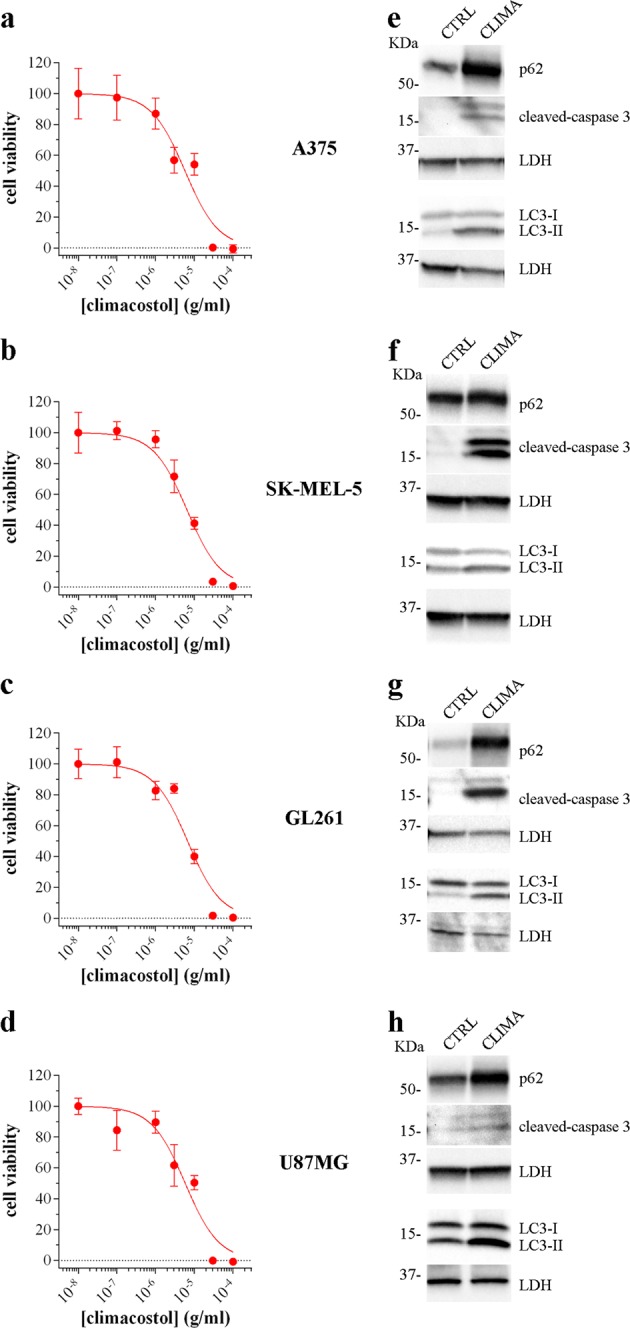
Climacostol effects in multiple tumour cell lines. MTT assay assessing the viability of A375 (**a**), SK-MEL-5 (**b**), GL261 (**c**), and U87MG (**d**) cells treated with increasing concentrations of climacostol for 24 h. Data are expressed by setting the absorbance of the reduced MTT in the absence of climacostol as 100%. The data points are representative of 8 independent experiments. Western blotting images of cleaved-caspase 3, LC3 and p62 expression in A375 (**e**), SK-MEL-5 (**f**), GL261 (**g**), and U87MG (**h**) cells cultured in the presence of 30 μg/ml climacostol (CLIMA) or control vehicle (CTRL) for 24. LDH was used as internal standard. Images are representive of 3 independent experiments

Human melanoma A375 and SK-MEL-5, murine glioma GL261 and human glioblastoma U87MG cells were treated for 24 h with climacostol. In agreement with previous results in multiple human and rodent cell lines^18,21–23^, climacostol caused a concentration-dependent reduction of cell viability with an E_max_ concentration value (nearly 100% inhibition) of ca. 30 μg/ml, as assessed by the MTT assay (Fig. 5a–d). Data also indicated that climacostol affects the viability with a comparable potency among cells, i.e., EC_50_ of 5.7, 6.4, 6.7 and 5.8 μg/ml for A375, SK-MEL-5, GL261 and U87MG cells, respectively. Similar results were obtained in B16-F10 cells as a control (Supplementary Fig. 1b)^23^. Climacostol treatment (24 h, 30 μg/ml) induced apoptosis and impaired autophagy in A375, SK-MEL-5, GL261 and U87MG cells since it increased the expression of cleaved-(active) executioner caspase 3 and led to an accumulation of LC3-II and p62 (Fig. 5e–h).

### Climacostol signalling regulating autophagy: p53-dependent and independent effects

**Fig. 6:**
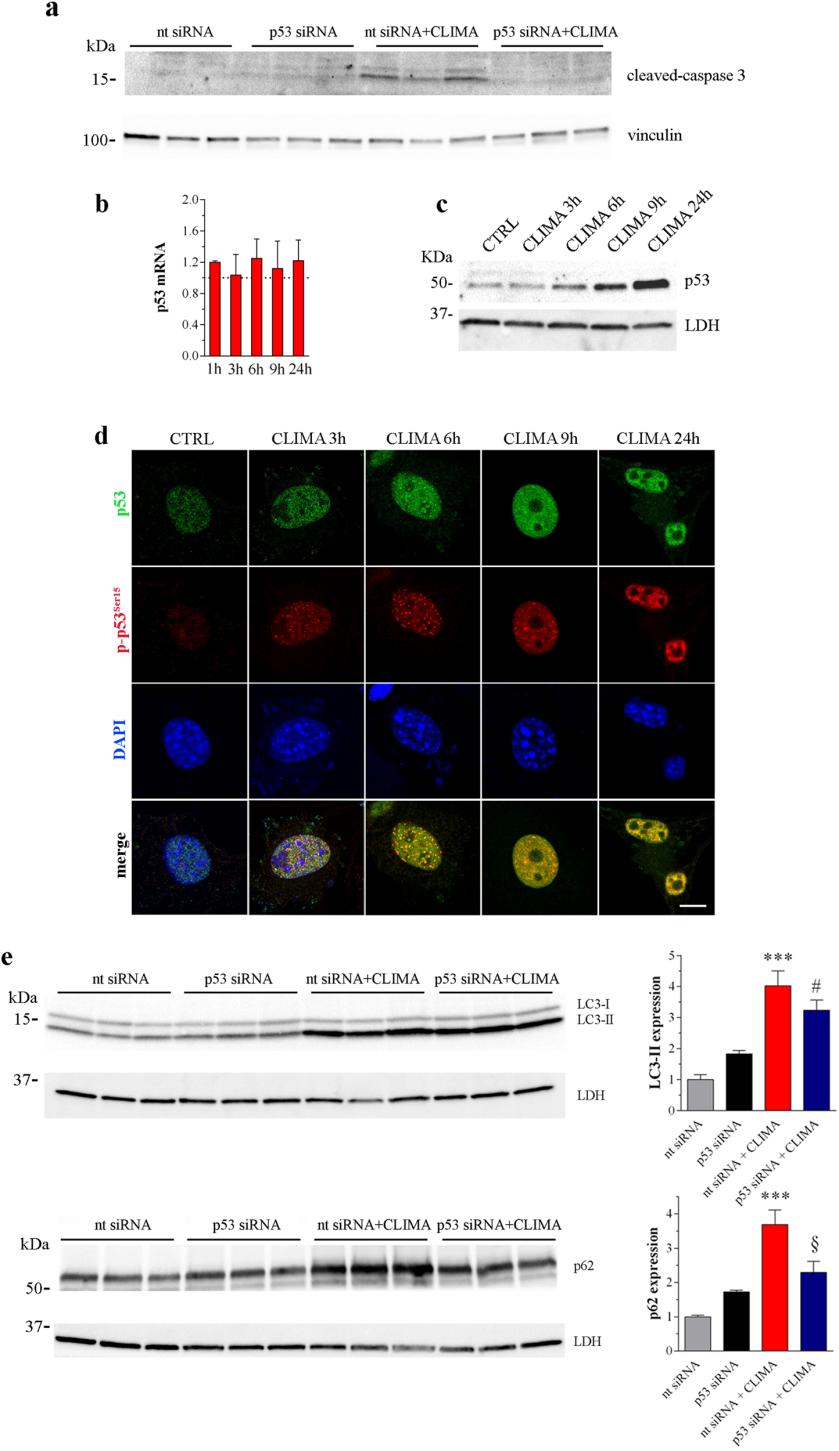
p53 is involved in the climacostol regulation of autophagy. **a** Western blotting images of cleaved-caspase 3 expression in B16-F10 cells transfected for 48 h with a p53-specific (p53 siRNA) or a non-targeting siRNA (nt siRNA), followed by vehicle or climacostol (CLIMA) treatment (24 h, 30 μg/ml). Vinculin was used as internal standard. **b**–**d** B16-F10 cells were cultured with 30 μg/ml CLIMA or control vehicle (CTRL) for increasing times. **b** mRNA levels of *p53* gene, as measured by real-time PCR. Results are expressed as fold change of control (dashed line), set as 1. **c** Western blotting images of p53 expression. LDH was used as internal standard. **d** Confocal immunofluorescence imaging of total p53 and p53 phosphorylated at Ser15 site (p-p53^Ser15^). Scale bar: 10 μm. DAPI was used for nuclei detection. **e** Western blotting images of LC3 and p62 expression in B16-F10 cells transfected for 48 h with a p53-specific (p53 siRNA) or a non-targeting siRNA (nt siRNA), followed by vehicle or CLIMA treatment (24 h, 30 μg/ml). LDH was used as internal standard. Right panels: densitometric analysis of LC3-II and p62 relative to their respective standard. Results are expressed as fold change of nt siRNA. ***p < 0.0001 relative to nt siRNA, #*p* < 0.05 relative to p53 siRNA, §*p* < 0.05 relative to nt siRNA + CLIMA. Images and data are representative of 3 independent experiments

Climacostol-induced pro-apoptotic effects in melanoma rely on the up-regulation of p53 that, in turn, activates the intrinsic programmed cell death pathway, including caspase 3^18,23^. This is confirmed by the analysis of cleaved-caspase 3 in B16-F10 cell transfected for 48 h with a p53-specific or a non-targeting siRNA^23^, followed by climacostol treatment (24 h, 30 μg/ml). Indeed, when the climacostol-dependent increase of p53 was silenced (Supplementary Fig. 2a) the activation of caspase 3 was abolished (Fig. 6a). The mRNA of *p53* did not change (Fig. 6b) while p53 protein clearly enhanced following climacostol exposure, with a detectable effect obtained at 6 h of treatment (Fig. 6c). Consistently, we detected a time-dependent accumulation of p53, almost completely localised in the nuclei of B16-F10 cells (Fig. 6d). The p53 protein phosphorylated at Ser15 site (p-p53^Ser15^), a modification responsible of p53 stability^25,26^, up-regulated as well in the presence of climacostol and p53/p-p53^Ser15^ staining was superimposable, thus indicating a post-translational effect on p53 induced by climacostol.

We then determined whether the role of climacostol on autophagic flux involved p53 signalling. The silencing of p53 perturbed climacostol effects on autophagic flux. LC3 lipidation in response to climacostol (24 h, 30 μg/ml) was still active (Fig. 6e). By contrast, p62 levels significantly decreased in p53 siRNA cells treated with climacostol reaching values comparable to control (Fig. 6e), despite climacostol inducing a sustained increase of the mRNA encoding *p62* in native cells (Supplementary Fig. 2b). This is consistent with a sustained autophagy turnover induced by climacostol in the absence of p53, thus suggesting that climacostol treatment simultaneously induces autophagosome formation and compromises autophagosome turnover, this latter via the up-regulation/phosphorylation of p53.

**Fig. 7:**
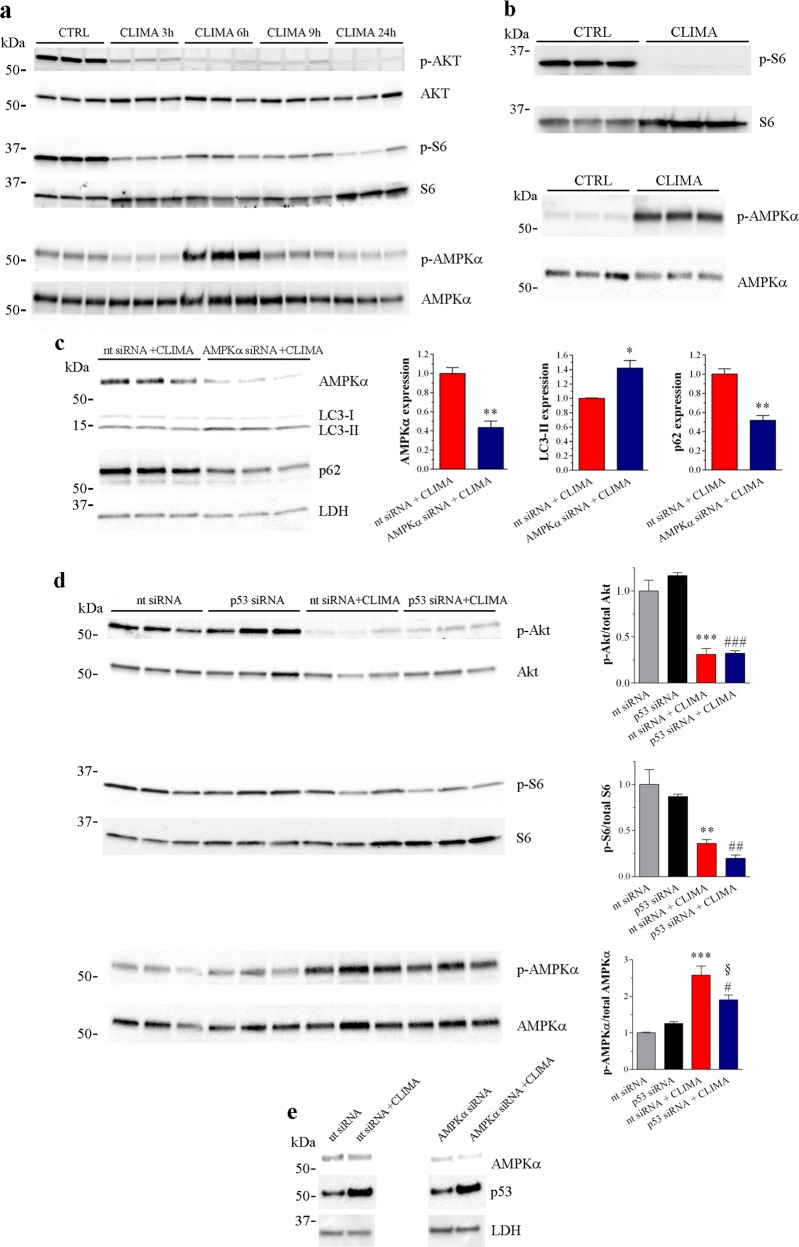
Autophagy signalling molecules involved in the climacostol regulation of autophagy. **a** B16-F10 cells were cultured with 30 μg/ml climacostol (CLIMA) or control vehicle (CTRL) for increasing times. Western blotting images of phosphorylated Akt, S6 and AMPKα. The total Akt, S6 and AMPK were used as internal standard. Images are representative of 6 independent experiments. **b** Western blotting images of phosphorylated S6 and AMPK in subcutaneous B16-F10 melanoma allografts excised from mice at day 16 of treatment (from day 0 - every 3–4 days) with 100 µl CLIMA (600 μg/ml) or CTRL. The total S6 and AMPK were used as internal standard. Images represent the results obtained from 6 animals per experimental group. **c** Western blotting images of AMPKα, LC3 and p62 expression in B16-F10 cells transfected for 48 h with an AMPKα-specific (AMPKα siRNA) or a non-targeting siRNA (nt siRNA), followed by CLIMA treatment (24 h, 30 μg/ml). LDH was used as internal standard. Right panels: densitometric analysis of AMPKα, LC3-II and p62 relative to their respective standard. Results are expressed as fold change of nt siRNA + CLIMA. Images and data are representative of 3 independent experiments. *p < 0.05 and **p < 0.005 relative to nt siRNA + CLIMA. **d** Western blotting images of phosphorylated Akt, S6 and AMPKα in B16-F10 cell transfected for 48 h with a p53-specific (p53 siRNA) or a non-targeting siRNA (nt siRNA), followed by vehicle or CLIMA treatment (6 h, 30 μg/ml). The total Akt, S6 and AMPKα were used as internal standard. Right panels: densitometric analysis of phosphorylated proteins relative to their respective standard. Results are expressed as fold change of nt siRNA. Images and data are representative of 3 independent experiments. **p < 0.005 and ***p < 0.0001 relative to nt siRNA; #*p* < 0.05, ##*p* < 0.005 and ###*p* < 0.0001 relative to p53 siRNA, §*p* < 0.05 relative to nt siRNA + CLIMA. **e** Western blotting images of AMPKα and p53 expression in B16-F10 cells transfected for 48 h with an AMPKα-specific (AMPKα siRNA) or a non-targeting siRNA (nt siRNA), followed by vehicle or CLIMA treatment (24 h, 30 μg/ml). LDH was used as internal standard. Images are representative of 3 independent experiments

To gain more mechanistic insights we evaluated different autophagy signalling molecules. The mammalian target of rapamycin (mTOR), when is activated by protein kinase B (PKB/Akt), drives the phosphorylation of autophagy proteins including S6^1–4^. The 5′-AMP-activated protein kinase (AMPK) can also impact on autophagy^1–4,35^. Within the temporal window of climacostol effects on autophagosomes, climacostol (30 μg/ml) triggered an early (3–6 h) decrease of Akt and S6 phosphorylation in B16-F10 cells which persisted over-time (Fig. 7a). In contrast, activated AMPKα substantially peaked at 6 h of treatment. Similar results were obtained in vivo, analysing melanoma allografts intra-tumour injected with 100 µl climacostol at 600 μg/ml or vehicle (control) every 3–4 days. The activity of S6 was lower in climacostol-injected tumours (at day 16 of treatment) while phosphorylated AMPKα increased (Fig. 7b), thus confirming that climacostol inhibits and stimulates mTOR and AMPK pathways, respectively. B16-F10 cells were then transfected for 48 h with an AMPKα-specific or a non-targeting siRNA, followed by climacostol treatment (24 h, 30 μg/ml). When the expression of AMPKα halved, the lipidation of LC3 by climacostol increased while p62 levels were significantly reduced (Fig. 7c), indicating the accumulation of autophagosomes via AMPK activation.

We next measured the phosphorylation of Akt/S6/AMPK after p53 silencing in cultured B16-F10 cells treated with 30 μg/ml climacostol for 6 h. In these conditions, the climacostol-induced up-regulation of p53 was abolished (Supplementary Fig. 2c). Notably, the decrease of Akt and S6 activity induced by climacostol was unchanged in non-targeting siRNA and p53 siRNA cells (Fig. 7d). On the contrary, the ablation of p53 significantly inhibited climacostol activation of AMPKα, that therefore is partially p53-dependent. AMPK regulates p53 acetylation and phosphorylation in cancers^36^. In B16 cells, the activation of AMPKα by the toxic natural compound vincristine is involved in p53 activation^37^. We found this not to be a key mechanism in our system since B16-F10 cell transfection with an AMPKα-specific siRNA did not affect p53 levels induced by climacostol (30 μg/ml) (Fig. 7e).

### Autophagy disruption and apoptotic cell death

**Fig. 8:**
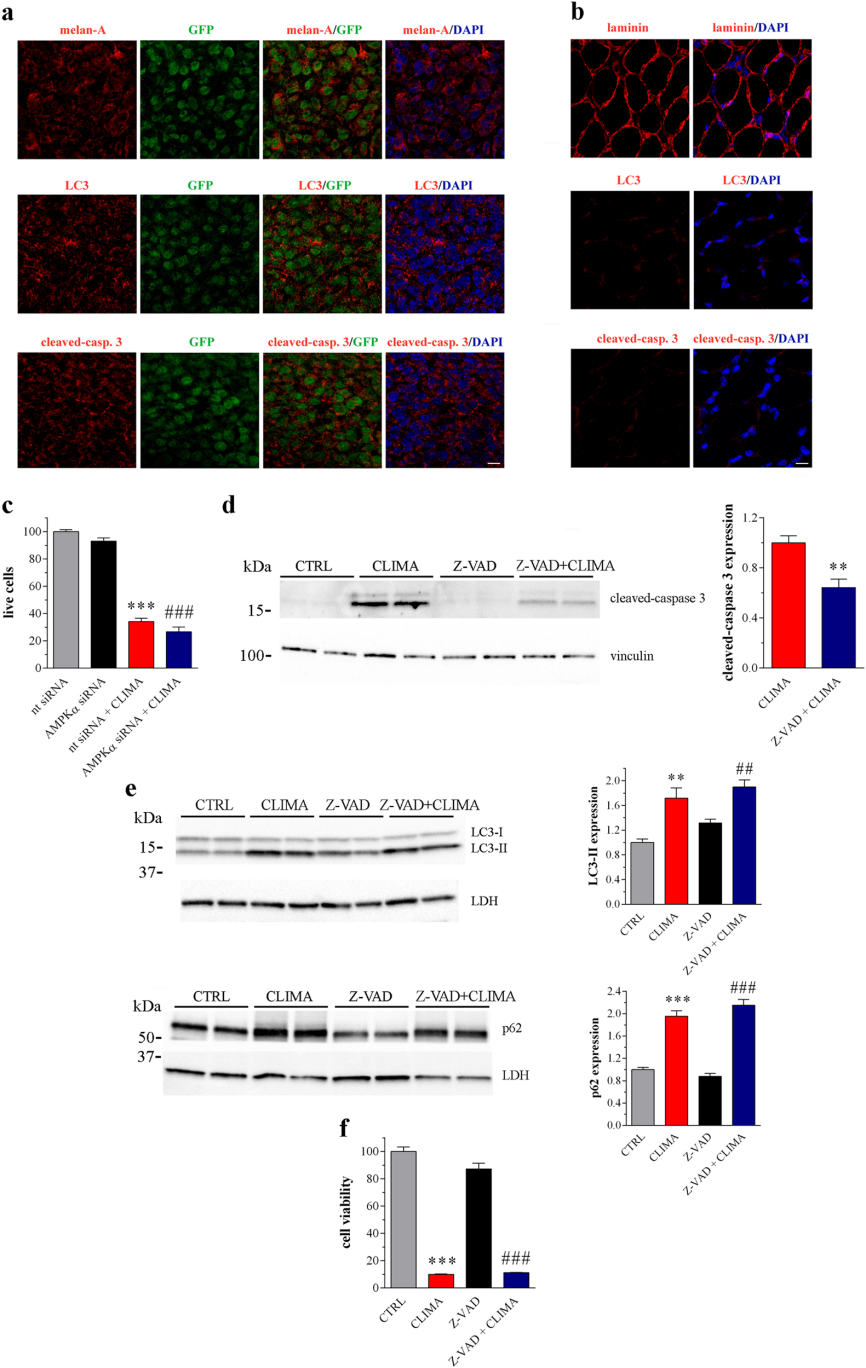
Autophagy/cell death events induced by climacostol. **a**, **b** GFP-expressing B16-F10 cells were injected into the tail vein of syngeneic mice the week before climacostol intraperitoneal treatment (4 mg/kg every 3–4 days for 2 weeks). Diaphragm, a skeletal muscle separating the thoracic/peritoneal cavities, was analysed by confocal microscopy 3 weeks after transplantation. **a** Confocal immunofluorescence imaging of melan-A, LC3 and cleaved-caspase 3 in tumour foci observed in the sections of diaphgram tissue. The signal of GFP (tumour cells) and DAPI (nuclei) was also detected. **b** Confocal immunofluorescence imaging of laminin, LC3 and cleaved-caspase 3 in diaphgram muscle. DAPI was used for nuclei detection. Scale bar: 50 μm. Images represent the results obtained from 5 animals. **c** Exclusion dye staining with trypan blue in B16-F10 cells (>5 × 10^5^ cells per experimental condition) transfected for 48 h with an AMPKα-specific (AMPKα siRNA) or a non-targeting siRNA (nt siRNA), followed by vehicle or climacostol (CLIMA) treatment (24 h, 30 μg/ml). Data are expressed by setting the number of living cells in control samples as 100%. Data are representative of 3 independent experiments. ***p < 0.0001 relative to nt siRNA; ###*p* < 0.0001 relative to AMPKα siRNA. **d - f** B16-F10 cells were cultured with 30 μg/ml CLIMA or control vehicle (CTRL) for 24 h, both in the absence and presence of the pan-caspase inhibitor Z-VAD-(OMe)-FMK (Z-VAD; 100 μM). **d** Western blotting images of cleaved-caspase 3 expression. Vinculin was used as internal standard. Right panel: densitometric analysis of cleaved-caspase 3 relative to the standard. Results are expressed as fold change of CLIMA. Images and data are representative of 3 independent experiments. **p < 0.005 relative to CLIMA.**e** Western blotting images of LC3 and p62 expression. LDH was used as internal standard. Right panels: densitometric analysis of LC3-II and p62 relative to their respective standard. Results are expressed as fold change of CTRL. Images and data are representative of 3 independent experiments. **p < 0.005 and ***p < 0.0001 relative to CTRL; ##*p* < 0.005 and ###*p* < 0.0001 relative to Z-VAD. **f** MTT assay assessing cell viability. Data are expressed by setting the absorbance of the reduced MTT in control samples as 100%. Data are representative of 8 independent experiments. ***p < 0.0001 relative to CTRL; ###*p* < 0.0001 relative to Z-VAD

The autophagy modulation in context with apoptosis was assessed in vivo. Climacostol at 2 and 4 mg/kg or vehicle (control) was injected intraperitoneally in mice every 3–4 days for 4 weeks, in line with the dosage used in the intra-tumour treatments. No animal died in the experimental or the control group; all mice appeared healthy and clinically normal, with no behavioural changes, suggesting the absence of systemic toxicity. The weight of climacostol-administered animals tended to increase, which was consistent with that of the control (Table 1). GFP-expressing B16-F10 cells (B16-GFP) were injected into the tail vein of syngeneic mice the week before climacostol intraperitoneal treatment (4 mg/kg every 3–4 days for 2 weeks) and diaphragm was analysed by fluorescence microscopy 3 weeks after transplantation. Tumour foci (Supplementary Fig. 2d), i.e., GFP and melan-A-positive cells, were clearly observed in diaphgram tissue (Fig. 8a). Melanoma cells expressed robust LC3 puncta and cleaved-caspase 3 staining (Fig. 8a) while these markers were almost undetectable in muscular (laminin-positive) cells (Fig. 8b).

**Table 1:** Body weight data

Week	CTRL	CLIMA	
		2 mg/kg	4 mg/kg
0	20.80 ± 0.28	20.78 ± 0.08	20.72 ± 0.23
1	22.27 ± 0.44	21.75 ± 0.52	21.39 ± 0.35
2	23.34 ± 0.58	22.79 ± 0.13	22.73 ± 0.58
3	24.29 ± 0.58	24.09 ± 0.54	24.36 ± 0.23
4	25.42 ± 0.57	25.08 ± 0.41	25.47 ± 0.48

The data points are expressed in grams and have been obtained from 3 animals per experimental group

*CTRL* control (vehicle), *CLIMA* climacostol

Autophagy and apoptosis cross-talk functions to maintain cellular homeostasis and respond to stress^38^. To test whether autophagy has a key role regulating cell fate in our system, we measured the cytotoxic effect of climacostol by exclusion dye staining with trypan blue in B16-F10 cells transfected with an AMPKα-specific or a non-targeting siRNA. As shown in Fig. 8c and Supplementary Fig. 2e, cell viability of AMPKα siRNA cells remained high. Climacostol (24 h, 30 μg/ml) displayed a similar high toxicity both in the presence of AMPKα or when its expression was knocked-down. A similar result was observed after compound C treatment (Supplementary Fig. 2f), a well-known inhibitor of AMPK.

Finally, B16-F10 cells were treated with climacostol (24 h, 30 μg/ml) both in the absence and presence of the pan-caspase inhibitor Z-VAD-(OMe)-FMK at 100 μM. The activation of caspase 3 induced by climacostol was reduced by Z-VAD-(OMe)-FMK (Fig. 8d). In contrast, caspase inhibition did not affect the accumulation of LC3-II and p62 (Fig. 8e) obtained after climacostol treatment thus clearly indicating that the activation of caspases is not associated with climacostol disruption of autophagic flux. Z-VAD-(OMe)-FMK administration did not rescue climacostol-induced reduction of MTT absorbance (Fig. 8f) thereby suggesting that climacostol effects on cell viability are not necessarily mediated by caspase-dependent mechanisms.

## Discussion

The cellular homeostatic process autophagy occurs at a basal level in all eukaryotic cells and may support cell survival or activate death pathways^10,11^. Many of the core autophagy genes found in humans are expressed in ciliated protozoa^39^. Alveolata (comprising single-celled ciliates, dinoflagellates and Apicomplexa) share with Opisthokonta (including Fungi and Animalia) a last common ancestor about 1.5 billion years ago. Notwithstanding the diversity and the evolutionary interval that separate these organisms, ciliates provide versatile molecular tools to determine autophagic pathway in mammals^6^. They synthesise a great variety of chemicals with biotech potential^18,40^. Our present findings show that climacostol, a pro-apoptotic natural compound produced by the ciliated protozoan *Climacostomum virens*, potently impairs autophagy. Climacostol, which has a chemical structure distinct from the current blockers of autophagic flux^8,15^, exerts a sustained accumulation of autophagosomes in tumour cells as the result of dysfunctional autophagic degradation. The analysis of B16-F10 allografts revealed a disruption of autophagy when melanomas were treated with intra-tumoural injections of climacostol. In addition, non-toxic doses of intraperitoneally administered climacostol reached diaphragm muscle selectively targeting transplanted melanoma cells, which thus showed impaired autophagy and were committed to die by apoptosis. In some cases climacostol toxicity was reported to be higher against tumour than non-tumour cells^18,21,23^ and not only cell death/apoptosis but also the impairment of autophagy was induced by climacostol in multiple cancer cell lines, i.e., human melanomas and murine/human glial tumour cells. Moreover, local delivery of climacostol inhibited melanoma growth thus inducing apoptosis and a significant improvement of animal survival^18,23^. These data indicate climacostol as a highly effective compound against a wide range of cancers, including those affecting humans. Many natural compounds exert pro- and anti-autophagic activity and thus may contribute to treatments of diverse human diseases^32,41^. Autophagy inhibition is a promising strategy and different agents disrupting autophagy are being evaluated in clinical trials for cancer treatment^14,15^. This study suggests that screening autophagy regulators from natural compounds might be an efficient methodology to identify novel autophagy inhibitors and lead compounds for cancer therapy.

Autophagy and apoptosis share key regulatory proteins, suggesting that the pathways regulating them are connected^38,42^. Since climacostol participates in the impairment of autophagy while inducing apoptosis, its action may be situated at the molecular crossroads regulating both autophagy and apoptosis. Our present data provide molecular ground and confirm this hypothesis. Firstly we demonstrated further that climacostol elicits apoptosis via p53^18,23^. Cimacostol activates p53, likely as a result of DNA damage, and its signalling, i.e., Noxa and PUMA^18,23^, inducing a quick up-regulation of p53 in the nuclei. This is not a change in gene transcription-mechanism but occur through effects on p53 stability at translational level^25,26^, as for instance the phosphorylation at Ser15 site. p53 is both a promoter and a suppressor of autophagy and these effects establish a p53-dependent cell fate^25,28^. We unravelled a double-edged role played by climacostol in either promoting autophagy, independently on p53 levels, or suppressing autophagosome turnover via the up-regulation of p53. In this way, i.e., coordinately inducing autophagosome accumulation and inhibiting the autophagic flux, more autophagic vacuoles may be accumulated in tumour cells. The natural compound bafilomycin A1 activates early stage of autophagy by downregulating mTOR pathway, and inhibits later stages of autophagy in hepatocellular carcinoma involving PUMA^43^. In addition, tetrandrine, which blocks autophagic flux and induces apoptosis in cancer cells, has been shown to induce a significant phosphorylation of AMPK^33^. In this respect, we provide evidence that the activation of autophagy by climacostol is likely due to an inhibition of mTOR signalling unrelated to p53; p53, via AMPKα activation, is nevertheless involved in the climacostol-induced impairment of autophagic process as AMPKα phosphorylation is under its control and the knock-down of AMPKα inhibited autophagosomes accumulation by climacostol. Accordingly, the activation of p53 increased the phosphorylation of AMPK and inhibited mTOR in cancer cells in which the completion of autophagy was inhibited^44^. In melanoma the activation of AMPK may induce accumulation of autophagosomes that are unable to be degraded when autophagosome clearance is inhibited^45^. Also, pro-inflammatory cytokines inhibit mTOR in β-cells, stimulate the AMPK axis and block autophagic flux^46^.

Autophagy may be either protective or toxic^10–12^ and AMPK may be connected with apoptosis regulation^47^. In melanoma the block of autophagy may aggravate or induce cell apoptosis^45,48–54^, due to excessive accumulation of autophagic vacuoles containing deleterious undegraded material. Climacostol is an efficient agent acting fast (between 3 h and 6 h) on autophagic flux resulting in autophagosome accumulation in the cytosol. Although dysfunctional autophagy in climacostol-treated cells occurs prior to detectable apoptosis^23^, the possibility that climacostol-induced cell death is downstream autophagy-related events is not supported by our data. The toxicity of climacostol against melanoma cells was not affected by the inhibition of AMPKα, thus the pro-apoptotic function of climacostol could not be attributed to the block of autophagosome turnover. Notably, climacostol disruption of autophagic flux is not associated with the activation of caspases and climacostol is also able to inhibit tumour cell viability without the involvement of caspases. These data indicate that climacostol effects on autophagy and apoptosis are two separate events, although both reflecting the upstream activation of p53. Noteworthy, they may act independently and in a redundant manner on life/death decisions of the cell. These observations provide a platform for future studies to explore the dual targeting of autophagy and apoptosis with cytotoxic agents acting on p53 for the killing of tumours that frequently develop chemoresistance due to protective autophagy^14,15^.

In essence, our study shows that climacostol impairs autophagy in tumours and suggests that the clinical potential of climacostol should be investigated further. We also generated valuable mechanistic insights identifying the p53-dependent increase of AMPK activity as the major responsible of autophagy disruption, although the mTOR pathway unrelated to p53-AMPK axis also plays a role. The up-regulation of p53 system is at the molecular crossroad regulating both the anti-autophagic action of climacostol and its role in the induction of apoptosis. In agreement with the promising paradigm of dual targeting of autophagy and apoptosis, different natural compounds have been shown to display anti-autophagic flux and pro-apoptotic effects in cancers^33,43,53,55–58^. Our findings suggest the efficacy of ciliate bioactive molecules, which have several intrinsic properties^18,40,59,60^, favouring their consideration in drug discovery and development.

## Materials and methods

### Climacostol

Chemically synthesised climacostol (C_15_H_22_O_2_, alkenyl resorcinol) was obtained as previously described^22^. The configuration of the double bond was assigned as a (*Z*)-based. Structure-activity studies have indicated that the C2–C3 unsaturation in the side chain plays a key role for the biological activity^18,22,23,61^. The (*Z*)-diastereomer is the active organic molecule, while the (*E*)-diastereomer is less active. The NMR spectroscopy of the climacostol obtained by our procedure allowed to determine that the content of (*Z*)-diastereomer was major than 99%, without contamination with the undesired (*E*)-diastereomer. Given that in the mixture of the two diastereomers the separation of the (*Z*)-diastereomer from its (*E*)-configuration was not possible through the common methods of separation, the natural toxin purified from cultures of *Climacostomum virens* was less active than synthetic climacostol. The latter one was then dissolved in absolute ethanol at 10 mg/ml stock, and stored in the dark at −20 °C until use. The stock solution of climacostol was diluted in phosphate buffered saline (PBS) (Euroclone, Pero, Italy) or in culture medium for in vivo injections and in vitro experiments, respectively. Where not indicated, the other reagents were purchased from Sigma-Aldrich (Saint Louis, MO, USA).

### Animals and cell cultures

Female C57BL/6 mice (8–12 weeks old) were purchased from Charles River Laboratories (Calco, Italy), housed in a regulated environment (23 ± 1 °C, 50 ± 5% humidity) with a 12 h light/dark cycle (lights on at 08.00 a.m.), and provided with food and water ad libitum. All studies were conducted in accordance with the Italian law on animal care N° 116/1992 and the European Communities Council Directive EEC/609/86. All efforts were made to reduce both animal suffering and the number of animals used.

Murine melanoma B16-F10, murine glioma GL261, human glioblastoma U87MG^23,54,62–64^, human melanoma A375 and SK-MEL-5 (obtained by the American Type Culture Collection) cell lines were cultured in Iscove’s supplemented with 10% heat-inactivated foetal bovine serum, glutamine (2 mM), penicillin/streptavidin (100 U/ml), 1% Hepes 1 M (Euroclone), pH 7.4. Cells were grown at 37 °C in a humidified atmosphere containing 5% CO_2_ (logarithmic growth phase, routine passages every 3 days). To create B16-GFP cells, B16-F10 cells were transfected with lentivirus pLVX-Puro (Takara Bio USA, Mountain View, CA, USA) encoding for EGFP, produced upon transfection of HEK-293T packaging cells with the lentiviral vector. After two cycles of infection cells were selected with puromycine (1 ug/ml) for 2 weeks in order to obtain a stable GFP-expressing cell line.

### RNA interference

Gene silencing of p53 and AMPKα in B16-F10 cells was performed as previously published^23,37^. Briefly, according to the manufacturer’s protocol, iBONI siRNA Pool (Riboxx, Radebeul, Germany) targeting mouse p53 (*trp53*) and AMPKα1/2 (Santa Cruz Biotechnology, Dallas,TX, USA) targeting mouse AMPKα (*prkaa1* and *prkaa2*) were mixed to Lipofectamine RNAiMax transfection reagent (Life Technologies, Monza, Italy). iBONI siRNA Pool negative control (Riboxx) and control siRNA-A (Santa Cruz Biotechnology) (non-targeting siRNAs) were also used. The mix was added to cultured B16-F10 cells at a siRNA concentration of 10–50 nM for 48 h.

### Animal handling and allograft tumour models

Using published protocols^18,23,54,62–65^, mice (weighing 18–21 g) received subcutaneous injections of 5 × 10^4^ cells B16-F10 in the lower-right flank. When the syngeneic implantation was established (usually 10 days after tumour cells inoculation) and the tumour was palpable (volume range between 15–30 mm^3^), mice were randomly assigned to one of the two experimental groups. In particular, transplanted mice received 100 μl intra-tumour injections of vehicle or climacostol (600 μg/ml, equivalent to ca. a concentration of 3 mg/kg dose) every 3–4 days. At day 16, mice were sacrificed, tumours removed and processed. In testing the toxicity of intraperitoneal injections of climacostol, mice were administered with dissolved drug (at 2 and 4 mg/kg) or vehicle every 3–4 days for 4 weeks. In another set of experiments, mice received 1 × 10^5^ B16-GFP cells into the tail vein^62^. The week after, climacostol was intraperitoneally injected every 3–4 days at 4 mg/kg. Two weeks after climacostol treatment, mice were sacrificed and the diaphragm skeletal muscle tissue removed.

### Real-time PCR

The analysis of mRNA expression was performed as previously described^18,23,62,66,67^. Briefly, total RNA from in vivo resected B16-F10 tumours and in vitro B16-F10 cells was extracted with the High Pure RNA Tissue Kit and the High Pure RNA Isolation Kit, respectively (Roche Applied Science, Mannheim, Germany), according to the manufacturer’s protocol. First-strand cDNA was generated from 1 μg of total RNA using iScript Reverse Transcription Supermix (Bio-Rad, Hercules, CA, USA). A set of primer pairs (Eurofins Genomics, Milano, Italy) was designed to hybridize to unique regions of the appropriate gene sequence (Supplementary Table 1). PCR was performed using SsoAdvanced Universal SYBR Green Supermix and the CFX96 Touch Real-Time PCR Detection System (Bio-Rad). The fold change was determined relative to the selected control sample after normalising to *Rpl32* (internal standard) by the formula 2^-ΔΔCT^.

### Fluorescence microscopy

As previously published^18,23,54,68^ in vivo resected B16-F10 tumours were immersion-fixed in 4% paraformaldehyde in 0.1 M phosphate buffer (PB), pH 7.4, for 3 h. The fixed tissue was transferred to 25% sucrose in PB. Tumour sections were cut at 10 μm with a cryostat, mounted onto positively charged slides and stored at −20 °C. Dissected diaphragm tissues^69,70^ were rapidly frozen and then cut at 10 μm with a cryostat, mounted onto positively charged slides and stored at −20 °C until use. Slides were then immersion-fixed in 4% paraformaldehyde in PB, pH 7.4, for 10 min. Sections were treated for 30 min at room temperature with 5% bovine serum albumin and 10% of normal goat serum (Life Technologies) in PB containing 0.5% Triton X-100. Overnight incubation was performed with one of the following rabbit primary antibodies: anti-LC3, anti-p62/SQSTM1 and anti-laminin A (Sigma-Aldrich), anti-melan-A (GeneTex, Irvine, CA, USA), anti-cleaved-caspase 3 (Cell Signaling Technology, Danvers, MA, USA)^71,72^ in PB containing 0.5% Triton X-100. For fluorescence detection, sections were stained with the appropriate Alexa Fluor secondary antibody (Life Technologies) in PB containing 0.5% Triton X-100 for 1.5 h and cover-slipped with Fluoroshield Mounting Medium containing DAPI (nuclei detection) (Abcam, Cambridge, UK). Incubation in secondary antibody alone was performed as a negative control. Images of resected tumours were acquired by a Zeiss Axioskop 2 plus microscope equipped with the Axiocam MRC photocamera and the Axiovision software (Carl Zeiss, Oberkochen, Germany). Images of diaphragm tissue were acquired by a Zeiss LSM 710 inverted confocal microscope.

Using published protocols^23,54,62^, in vitro B16-F10 cells cultured in 120-mm coverslips were fixed in 4% paraformaldehyde in 0.1 M PB, pH 7.4, for 10 min and overnight stained with rabbit anti-LC3, anti-p62/SQSTM1 and anti-phospho-p53 (Ser15), and mouse anti-p53 (Cell Signaling Technology) primary antibodies. Cells were also stained with the appropriate Alexa Fluor secondary antibodies in PB containing 0.5% Triton X-100 for 1 h and cover-slipped in a ProLong Gold Antifade Mountant (Life Technologies), stained with fluorescein phalloidin (cytoskeleton detection) (Life Technologies) and DAPI (Sigma-Aldrich). Slides were analysed using a DMI4000 B automated inverted microscope equipped with a DCF310 digital camera (Leica Microsystems, Wetzlar, Germany). Confocal imaging was performed with a Leica TCS SP5 AOBS microscope system. Image acquisitions were controlled by the Leica LAS AF software.

To perform quantitative analysis for LC3 and p62 immunostaining^72–74^, images were converted to grayscale and normalised to background using Adobe Photoshop software (Adobe Systems, Mountain View, CA, USA). Mean gray levels were then measured in the selected tumour area or cells.

### Western blotting

Using published protocols^18,23,54,62,75,76^, in vivo resected B16-F10 tumours and human or murine cancer cell lines were homogenised in RIPA lysis buffer, supplemented with a cocktail of protease and phosphatase inhibitors (cOmplete and PhosSTOP; Roche Diagnostics, Milano, Italy). Equal amounts of proteins were separated by 4–20% SDS-polyacrylamide gel electrophoresis (Criterion TGX Stain-free precast gels and Criterion Cell system; Bio-Rad) and transferred onto nitrocellulose membrane using a Bio-Rad Trans-Blot Turbo System. When indicated, the membranes were probed using the rabbit anti-LC3, anti-p62/SQSTM1, anti-cleaved-caspase 3, anti-phospho-Akt (Ser473), anti-phospho-S6 (Ser240/244), anti-phospho-AMPKα (Thr172) and mouse anti-p53 (Cell Signaling Technology)^71,72^ primary antibodies. After the incubation with the appropriate horseradish-peroxidase-conjugated secondary antibody (Cell Signaling Technology), bands were visualised using the Clarity Western ECL substrate with a ChemiDoc MP imaging system (Bio-Rad). To monitor for potential artefacts in loading and transfer among samples in different lanes, the blots were routinely treated with the Restore Western Blot Stripping Buffer (ThermoFisher Scientific, Waltham, MA, USA) and re-probed with the mouse anti-vinculin (Sigma-Aldrich) or goat anti-LDH-A (Santa Cruz Biotechnology). Primary antibodies, i.e., rabbit anti-Akt and anti-AMPKα, and mouse anti-S6 (Cell Signaling Technology), that recognize the protein independently of its phosphorylation state, were also used in re-probing experiment for normalisation purposes. When appropriated, bands were quantified for densitometry using the Bio-Rad Image Lab software.

### Transmission electron microscopy

The collected B16-F10 cells were stored overnight at 4 °C in a fixative solution containing 2.5% (v/v) glutaraldehyde and 2% (v/v) paraformaldehyde in 0.1 M cacodylate buffer, pH 7.2. Fixed cells were washed in cacodylate buffer and post-fixed with 2% (v/v) osmium tetroxide in 0,1 M cacodylate buffer, pH 7.2 for 2 h at 4 °C. Samples were washed in the same buffer and dehydrated through an ascending series of ethanol and embedded in LRWhite resin (Electron Microscopy Science, PA, USA). For ultrastructural observations at least 20 ultra-thin sections (60–90 nm) were obtained using a Reichert Ultracut ultramicrotome equipped with a diamond knife (Leica Microsystems). Ultra-thin sections were collected on copper grids, stained with uranyl acetate and lead citrate, and observed with a 1200 EXII electron microscope (Jeol, Tokyo, Japan). Micrographs were captured by the SIS VELETA CCD camera equipped with iTEM software (Olympus, Tokyo, Japan).

### MTT and Trypan blue viability assay

Cell viability on human or murine cancer cell lines was determined by MTT assay using published protocols^59,60,62,67,77–79^. MTT absorbance was quantified spectrophotometrically using a Glomax Multi Detection System microplate reader (Promega, Milano, Italy). B16-F10 cells were also stained with trypan blue (Bio-Rad) and the amount of living cells was determined using a Bio-Rad TC10 Automat Cell Counter. Cells were visualised using a Leica DMI4000 B automated inverted microscope equipped with a DCF310 digital camera.

### mRFP-GFP-LC3 assay

B16-F10 cells were plated on 14 mm coverslips coated with poly-D-lysine and then cultured for 24 h. Cells were then transiently transfected with tandem fluorescent mRFP-GFP-LC3 plasmid^80^, kindly provided by Dr. Pura Muñoz-Cánoves (Pompeu Fabra University, Barcelona, Spain), using Lipofectamine LTX and Plus Reagent (Life Technologies). Three hours following transfection at 37 °C, two-thirds of the media is replenished with fresh media. After drug treatments, cells were washed once in PBS and fixed with 4% paraformaldehyde (in PBS) for 15 min at room temperature. After washing, coverslips were mounted on glass slides with ProLong Gold Antifade Mountant with DAPI and analysed using a Carl-Zeiss LSM 710 inverted confocal microscope. The number of autophagosomes (number of yellow puncta per cell) and autolysosomes (number of red puncta per cell) was quantificated per cell, and at least 100 cells for each experiment were included.

### Statistics

Statistical significance of raw data between the groups in each experiment was evaluated using unpaired Student’s *t* test (single comparisons) or one-way ANOVA followed by the Newman-Keuls post-test (multiple comparisons). EC_50_ (the concentration producing half the maximum effect) and *E*_max_ concentration (producing the maximum effect) were determined by non-linear regression curve analysis of the concentration-effect responses. Potency values among concentration-response curves were compared with the F-test. Data belonging from different experiments were represented and averaged in the same graph. The GraphPad Prism software package (GraphPad Software, San Diego, CA, USA) was used. The results were expressed as means ± SEM of the indicated n values.

## Supplementary information

Supplementary Figure 1

Supplementary Figure 2

Supplementary Figure Legends

Supplementary Table 1
