# Supplementary figures and images for: Dysfunctional autophagy induced by the pro-apoptotic natural compound climacostol in tumour cells
Source: Cell Death Dis. 2018 Dec 19;10(1):10. doi: 10.1038/s41419-018-1254-x (PMC6315039; doi:10.1038/s41419-018-1254-x)

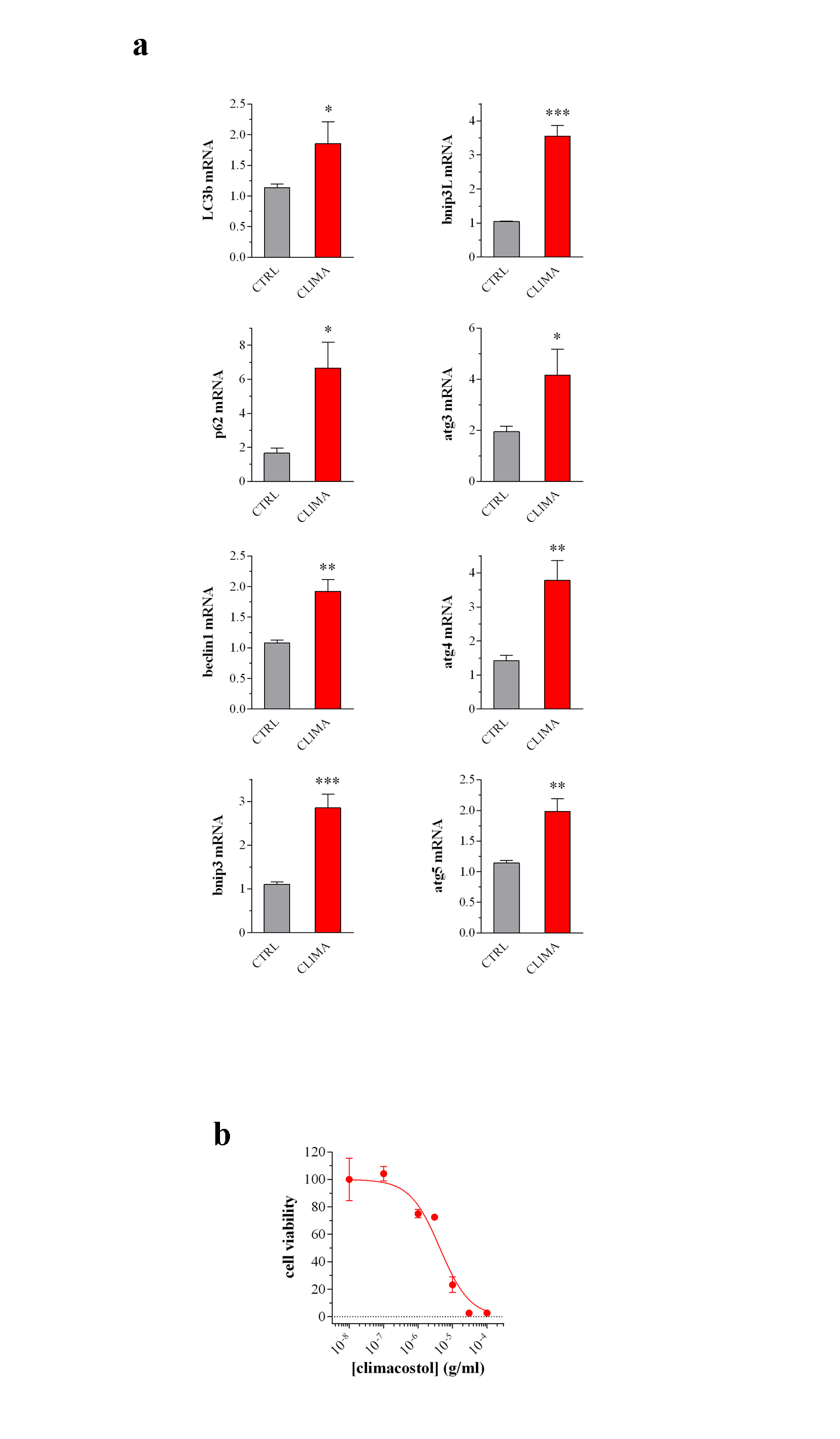

Supplement: Supplementary file 1 — Supplementary Figure 1 [file 41419_2018_1254_MOESM1_ESM.tif]

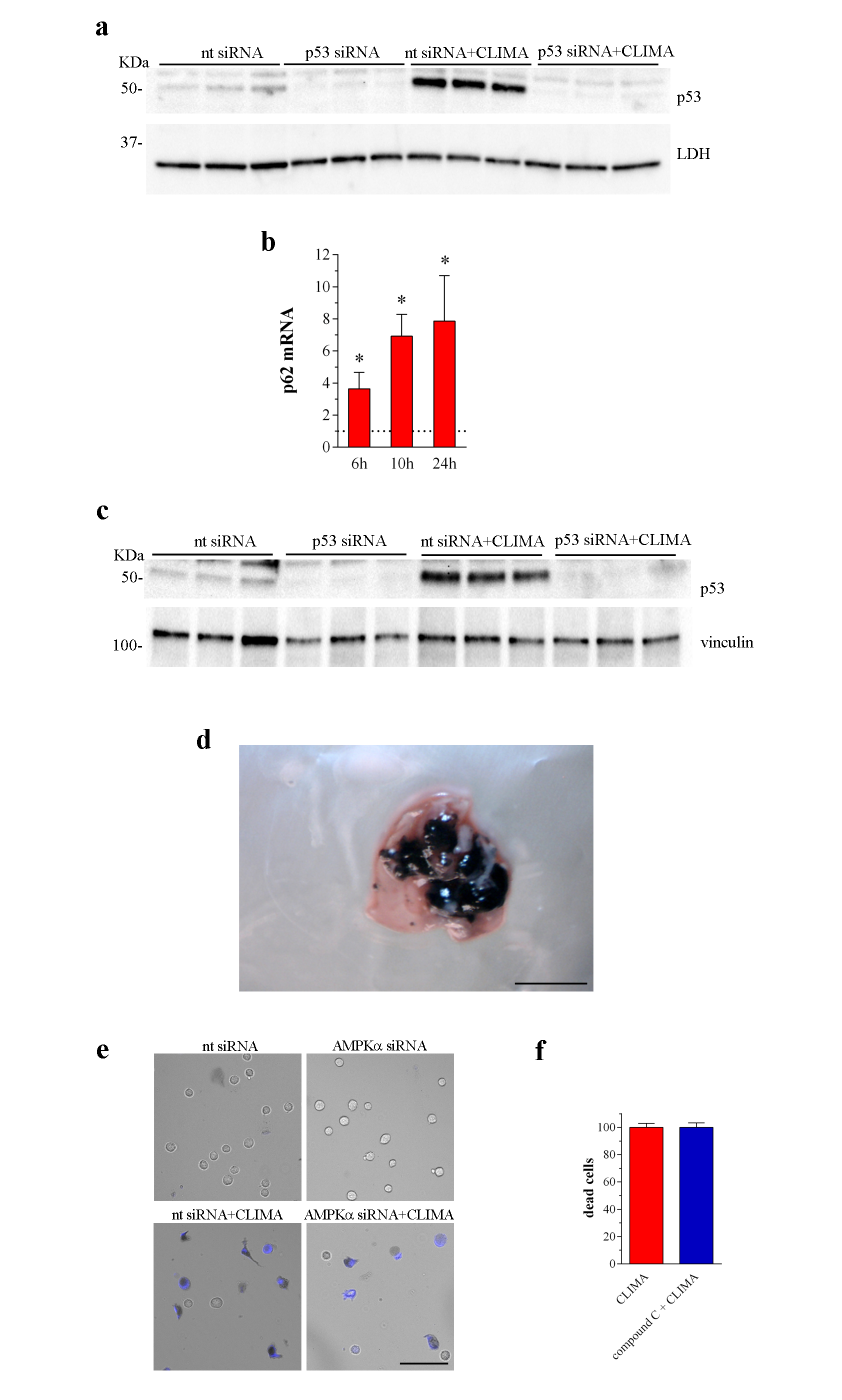

Supplement: Supplementary file 2 — Supplementary Figure 2 [file 41419_2018_1254_MOESM2_ESM.tif]
