# Supplementary material for: Dysfunctional autophagy induced by the pro-apoptotic natural compound climacostol in tumour cells
Source: Cell Death Dis. 2018 Dec 19;10(1):10. doi: 10.1038/s41419-018-1254-x (PMC6315039; doi:10.1038/s41419-018-1254-x)
Supplement: Supplementary file 3 — Supplementary Figure Legends [file 41419_2018_1254_MOESM3_ESM.docx]

**Supplementary Figure 1.** **a** Subcutaneous B16-F10 melanoma allografts were excised from mice at day 16 of treatment (from day 0 - every 3-4 days) with 100 µl climacostol (CLIMA; 600 μg/ml) or control vehicle (CTRL). mRNA levels of autophagy genes were measured by real-time PCR. Results are expressed as fold change of CTRL. *p < 0.05, **p < 0.005 and ***p < 0.0001 relative to CTRL. Data represent the results obtained from 6 animals per experimental group. **b** MTT assay assessing the viability of B16-F10 cells treated with increasing concentrations of climacostol for 24 h. Data are expressed by setting the absorbance of the reduced MTT in the absence of climacostol as 100%. The data points are representative of 8 independent experiments.

**Supplementary Figure 2. a** Western blotting images of p53 expression in B16-F10 cells transfected for 48 h with a p53-specific (p53 siRNA) or a non-targeting siRNA (nt siRNA), followed by vehicle or climacostol (CLIMA) treatment (24 h, 30 μg/ml). LDH was used as internal standard. Images are representative of 3 independent experiments. **b** B16-F10 cells were cultured with 30 μg/ml climacostol or control vehicle for increasing times. mRNA levels of p62 gene was measured by real-time PCR. Results are expressed as fold change of control (dashed line), set as 1. *p < 0.05 relative to respective control. Data represent the results obtained from 6 independent experiments. **c** Western blotting images of p53 expression in B16-F10 cell transfected for 48 h with a p53-specific (p53 siRNA) or a non-targeting siRNA (nt siRNA), followed by vehicle or CLIMA treatment (6 h, 30 μg/ml). LDH was used as internal standard. Images are representative of 3 independent experiments. **d** GFP expressing B16-F10 cells were injected into the tail vein of syngeneic mice the week before climacostol intraperitoneal treatment (4 mg/kg every 3-4 days for 2 weeks). Typical photographs of diaphragm tissue with melanoma tumour foci, excised from mice 3 weeks after transplantation. Scale bar: 1 cm. Image represent the results obtained from 5 animals. **e** Images depicting the exclusion dye staining with trypan blue in B16-F10 cells transfected for 48 h with an AMPKα-specific (AMPKα siRNA) or a non-targeting siRNA (nt siRNA), followed by vehicle or CLIMA treatment (24 h, 30 μg/ml). Scale bar: 50 μm. Images are representative of 3 independent experiments. **f** Exclusion dye staining with trypan blue in B16-F10 cells treated with CLIMA (24 h, 30 μg/ml) in the absence or in the presence of compound C (3 h pre-treatment, 10 μM). Data are expressed by setting the number of dead cells induced by CLIMA as 100%. Data are representative of 3 independent experiments.
