# Supplementary material for: Dysfunctional autophagy induced by the pro-apoptotic natural compound climacostol in tumour cells
Source: Cell Death Dis. 2018 Dec 19;10(1):10. doi: 10.1038/s41419-018-1254-x (PMC6315039; doi:10.1038/s41419-018-1254-x)
Supplement: Supplementary file 4 — Supplementary Table 1 [file 41419_2018_1254_MOESM4_ESM.docx]

**Supplementary Table 1** **Primer pairs designed for real-time PCR analysis**

| **Name** | **Gene** | **Primer sequence** |
| --- | --- | --- |
| LC3b | *map1lc3b* | F: 5’-CACTGCTCTGTCTTGTGTAGGTTG-3’  R: 5’-TCGTTGTGCCTTTATTAGTGCATC-3’ |
| p62 | *sqstm1* | F: 5’-GAAGCTGCCCTCTACCCACA-3’  R: 5’-AGAAACCCATGGACAGCATC-3’ |
| beclin1 | *becn1* | F: 5’-TGAATGAGGATGACAGTGAGCA-3’  R: 5’-CACCTGGTTCTCCACACTCTTG-3’ |
| bnip3 | *bnip3* | F: 5’-TTCCACTAGCACCTTCTGATGA-3’  R: 5’-GAACACGCATTTACAGAACAA-3’ |
| bnip3L | *bnip3l* | F: 5’-TTGGGGCATTTTACTAACCTTG-3’  R: 5’-TGCAGGTGACTGGTGGTACTAA-3’ |
| atg3 | *atg3* | F: 5’-CGGTCCTCAAGGAATCAAAA-3’  R: 5’-TAGCTTTGCAGGCTTCCACT-3’ |
| atg4 | *atg4b* | F: 5’-ATTGCTGTGGGGTTTTTCTG-3’  R: 5’-AACCCCAGGATTTTCAGAGG-3’ |
| atg5 | *atg5* | F: 5’-AGCAGCTCTGGATGGGACTGC-3’  R: 5’-GCCGCTCCGTCGTGGTCTGA-3’ |
| p53 | *trp53* | F: 5’-CACGTACTCTCCTCCCCTCAAT-3’  R: 5’-AACTGCACAGGGCACGTCTT-3’ |
| rpl32 | *rpl32* | F: 5’-TTAAGCGAAACTGGCGGAAAC-3’  R: 5’-TTGTTGCTCCCATAACCGATG-3’ |

F: forward, R: reverse
